# Diffusion Smart-seq3 of breast cancer spheroids to explore spatial tumor biology and test evolutionary principles of tumor heterogeneity

**DOI:** 10.1038/s41598-024-83989-x

**Published:** 2025-01-30

**Authors:** Antony Cougnoux, Loay Mahmoud, Per A. Johnsson, Alper Eroglu, Louise Gsell, Jakob Rosenbauer, Rickard Sandberg, Jean Hausser

**Affiliations:** 1 Department of Cell and Molecular Biology, Karolinska Institutet, Stockholm, Sweden; 2 Science for Life Laboratory, Stockholm, Sweden

**Keywords:** Systems biology, Systems analysis

## Abstract

Combining 3D cultures such as tumor spheroids and organoids with spatial omics holds great potential for tissue biology and cancer research. Yet, this potential is presently limited by technical and financial challenges of spatial omics methods and 3D cultures. To address this, we combine dye diffusion, the Smart-seq3xpress protocol for deep single-cell gene expression profiling, and dedicated probabilistic inference methods into diffusion Smart-seq3 (Smart-seq3D), to reveal the transcriptome of single cells along with their position along the core-periphery axis of spheroids. Applying Smart-seq3D to triple-negative breast tumor spheroids identifies thousands of spatial genes and reveals continuous, ungated spatial gene expression. Spatial gene and pathway expression patterns suggest biologies specific to spheroid regions, which we validate by immunostainings and pharmacological interventions. We use the Smart-seq3D data to test evolutionary principles of spatial tumor heterogeneity. Finally, we characterize aspects of tumor heterogeneity captured by 3D spheroids that are missing from 2D cultures but found in tumors *in vivo*. Smart-seq3D can offer a cost-efficient approach to explore how cells adapt their transcriptome to different micro-environments, reveal spatial determinants of drug resistance and could serve to characterize spatial interactions between cancer and stromal/immune cells in 3D co-cultures.

**Supplementary Information:**

The online version contains supplementary material available at 10.1038/s41598-024-83989-x.

## Introduction

Combining *in vitro* culture methods that mimic the 3D structure of tissues with spatial omics has the potential to transform our understanding of human tissue biology in health and disease. For example, organoids from pluripotent or adult stem cells self-organize as 3D structures that are highly similar to - and in some cases, histologically indistinguishable from - human organs^1^. Organoids mimic the development and regeneration of human organs which can be difficult to replicate in animal models. They can be used to research the mechanisms underlying these processes, as well as how human organ biology is perturbed by genetic disorders and infections. They represent a platform to discover and test new drugs specific to human biology^1^.

In cancer research, three-dimensional *in vitro* models of tumors called spheroids are receiving increasing attention. Compared to the common 2D culture of cancer cell lines, 3D spheroids feature cell-cell interactions and spatial heterogeneity similar to avascular tumors^2,3^. In particular, cells on the spheroid periphery proliferate while cells located in the core undergo necrosis due to lack of oxygen and nutrients. The local lack of oxygen and nutrients in tumors supports resistance to anti-cancer drugs^4,5^, which makes tumor spheroids an attractive platform to screen and test novel compounds. Three dimensional cultures also allows to dissect interactions of cancer cells with host cells - immune, stroma, endothelia - and bacteria^6–8^. Compared to animal models, 3D spheroids are faster, more controllable experimentally, higher-throughput, less subject to ethical considerations of animal experiments, and minimize the risk that findings fail to translate from animal to human.

Since organoids and spheroids can model the spatial architecture of organs and tissues, there is scientific benefit to profile them by spatial omics assays. Spatial omics assays determine the position of individual cells along with the abundance of dozens to thousands of proteins (MIBI^9^, CycIF/4i/HIFI^10–12^, Codex phenocycler^13^, spatial proteomic^14^) and the expression of hundreds or thousands of genes in each cell (ISS/Xenium^15^, MERFISH^16^, Visium^17^, Slide-tag^18^, smFISH + scRNAseq ^19^) of a tissue section. The data can serve to discover histological niches and neighborhoods that structure these tissues, illuminate the biology of developing and healthy tissue, and unravel disease etiology^20^. In the context of tumor spheroids, these technologies could for example reveal the spatial basis of the transcriptional heterogeneity of tumors, as well as transcriptional determinants of drug resistance to target in therapy.

There are limitations, however, to applying existing spatial omics techniques to tumor spheroids. Approaches based on *in situ* RNA hybridization are perhaps best suited for spatial profiling of spheroids due to their ability to quantify hundreds to thousands of genes with single-cell resolution in 3D^21^. Yet, segmenting RNA molecules into single cells remains a challenge. Another difficulty is that spheroids need to be positioned on a single plane to be sectioned, which is technically challenging due to their small size (~ 500 μm) compared to tissue sections (~ 1 cm). These assays are costly which limits their accessibility to the research community.

To overcome these limitations, dye diffusion can be combined with fluorescence-activated cell sorting (FACS). Adding a dye to the culture medium and allowing partial diffusion into the spheroid labels cells according to their position within the spheroid, with higher dye intensity at the periphery and lower dye intensity at the core^22–24^. Markers of cells with high vs. low dye intensity can then be compared by flow cytometry^22,24^ or cells sorted for single-cell transcriptomics^23,25^ to compare markers and gene expression of cells from the periphery vs. the core of spheroids. Doing so revealed that chromatin remodeling drives the spatial metabolic heterogeneity of spheroids of lung cancer cell lines^23^, and that cells at the periphery of spheroids of fibrocystic breast cell line express transcriptional programs associated to invasion and metastasis^25^.

Current dye diffusion-based approaches to spatial single-cell transcriptomics of spheroids have limitations of spatial resolution and transcriptome profiling depth. Their limited spatial resolution comes from the requirement to sort cells into bins of dye intensity prior to performing single-cell transcriptomics. For example, sorting cells according to high vs. low dye intensity, we can identify genes expressed more at the periphery and in the core. Yet, we miss genes whose expression peaks in the intermediate regions of spheroids in between the core and the periphery. In addition, single-cell profiling of sorted cells by emulsion-based methods have limited RNA molecule recovery rate, typically capturing less than 10% of a cell’s transcriptome^26^. This prioritizes the profiling of the highest expressed genes - ribosomal proteins, spliceosome, glycolysis^27^ - with the risk to miss the finer-grained biology encoded in the remaining 90% of the transcriptome.

**Fig. 1:**
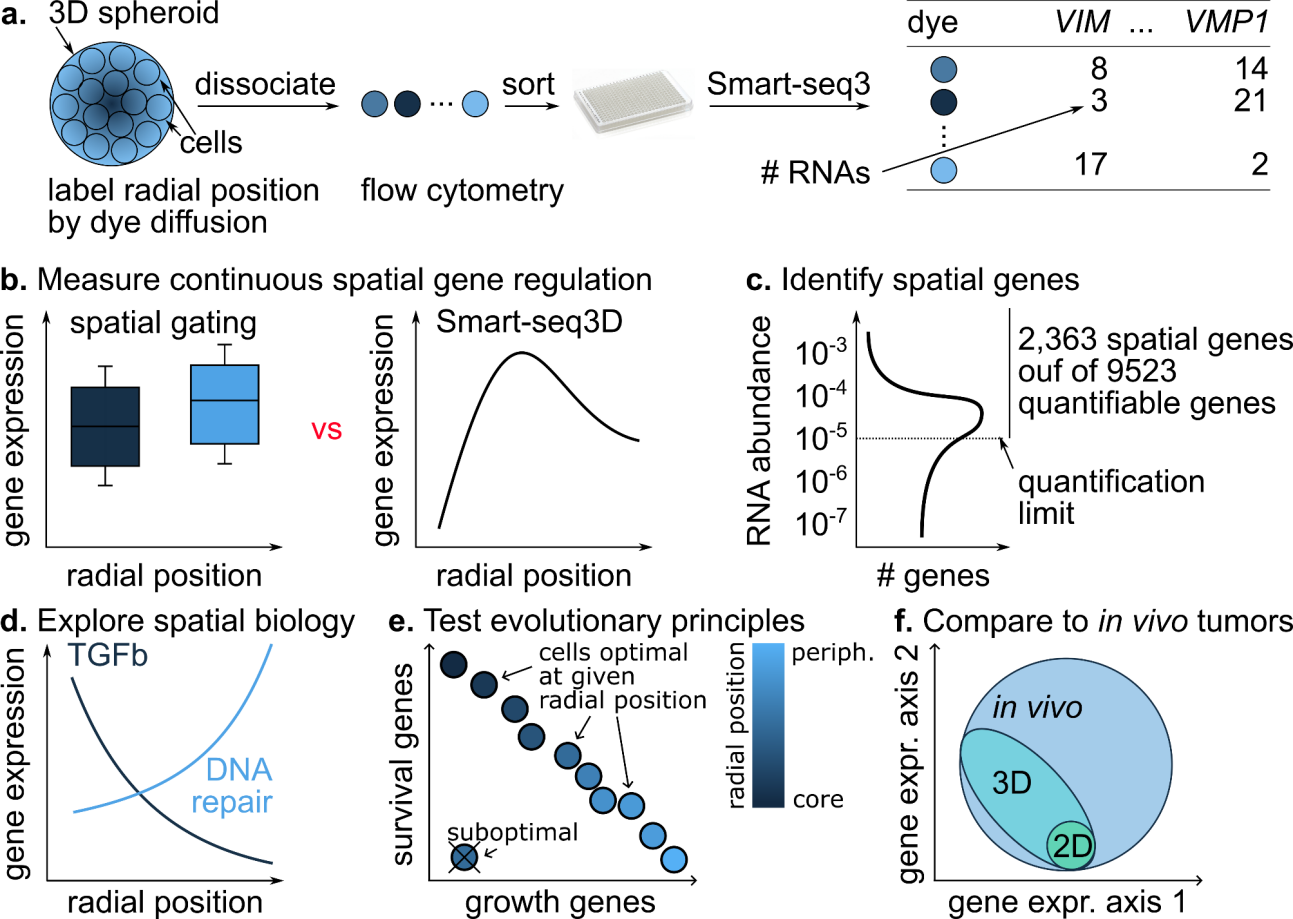
Overview of the present study. **(a)** Smart-seq3D integrates dye diffusion, Smart-seq3xpress, and probabilistic inference to map single-cell transcriptomes in tumor spheroids. **(b)** Smart-seq3D reveals un-gated, continuous gene expression patterns. **(c)** Deep single-cell transcriptomics by Smart-seq3xpress identified 2363 spatial genes among 9523 genes with an average of at least one RNA copy per cell. Applying Smart-seq3D to tumor spheroids allowed **(d)** exploring spatial tumor biology, **(e)** testing evolutionary principles of tumor heterogeneity and **(f)** revealing aspects of transcriptional heterogeneity of in vivo tumors captured by 3D spheroids.

Here, we combine dye diffusion, the Smart-seq3xpress protocol which provides among the deepest single-cell transcriptome coverage^28^, and a dedicated statistical inference approach into a new method for spatial, single-cell transcriptomics of spheroids which we call diffusion Smart-seq3 (in short: Smart-seq3D, Fig. 1). We apply Smart-seq3D to triple-negative breast tumor (TNBC) spheroids. Smart-seq3D boosts spatial resolution and transcriptome profiling depth to identify thousands of potential spatial genes with diverse spatial patterns, including patterns that cannot be detected by a two-bin sort-and-sequence approach. We infer the biology of the different spheroid areas by pathway analysis and validate the findings by immunostainings and pharmacological interventions. We illustrate potential applications of Smart-Seq3D: testing evolutionary principles of spatial tumor heterogeneity, and identifying aspects of intra-tumor heterogeneity captured by 3D spheroids that are missing from 2D cultures but found in tumors *in vivo* (Fig. 1).

## Results

### Smart-seq3D of tumor spheroids reveals the deep transcriptome of single cells along with their position along the core-periphery axis of spheroids

Adding a dye in the growth medium of spheroids and allowing the dye to partially diffuse into spheroids preferentially labels cells at the spheroid periphery, thereby distinguishing cells at the spheroid periphery from those in their core^22–24^.

We applied this approach to spheroids of MDA-MB-231 cells, an adherent triple-negative breast cancer (TNBC) cell line^29^. TNBC is a type of breast cancer negative for the Her2 and both the estrogen and progesterone receptors. Among breast cancer types, TNBC has worst prognosis and presently lacks effective treatment.

**Fig. 2:**
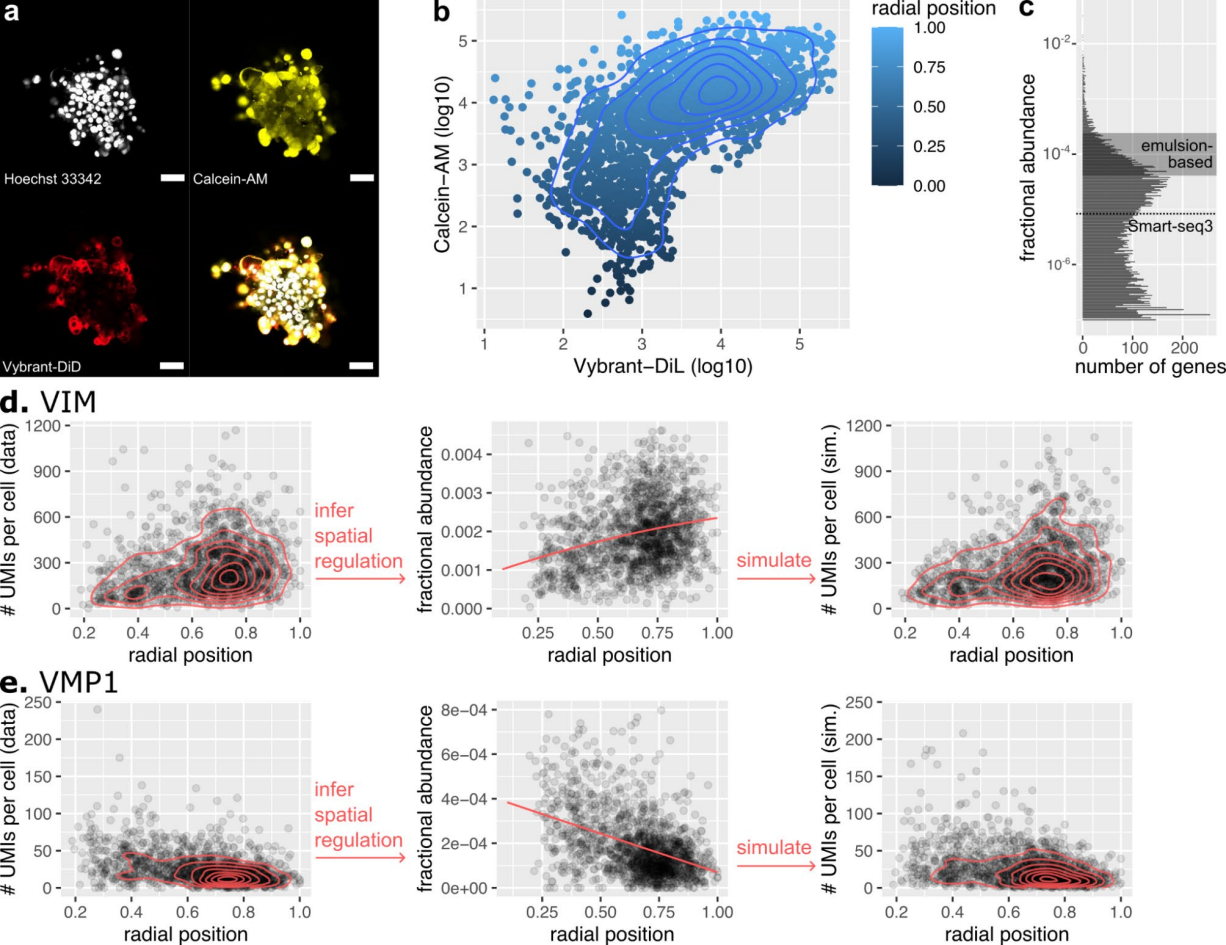
**(a)** Incomplete diffusion of MDA-MB-231 spheroids by Vybrant-DiL and Calcein-AM labels cells according to their position on a core-periphery axis. Representative immunofluorescence microscopy image of a spheroid incubated for 2 h with 10µM of Vybrant-DiL and Calcein-AM before clearing by Ominpaque. Scale-bar: 100 μm **(b)** The Calcein-AM intensity from flow cytometry is used to estimate the radial position of cells on a core-periphery axis. **(c)** Deep transcriptomics by Smart-seq3D can quantify expression of lower expressed genes in individual cells compared to emulsion-based methods. Sequencing *n* UMIs per cell, only genes with fractional abundance larger than 1/*n* are expected to be quantifiable (i.e. yield at least 1 UMI per cell) without borrowing information across cells by computational methods. Histogram: for each gene, fractional mRNA abundance was computed by summing up UMIs from all 1527 cells and normalizing by the total number of UMIs from all cells so that UMIs from all genes sum up to 1. **d-e.** From UMI counts in cells of different radial positions, a probabilistic model infers spatial gene regulation along with gene specific noise parameters. Simulating UMI counts from the model faithfully recapitulates the spatial distribution of UMIs, validating the ability of the probabilistic model to infer spatial regulation. Shown are the peripherally-expressed VIM gene (d) and the core-expressed VMP1 gene (e). Dots: single cells. Contours: density of cells from two-dimensional kernel estimator.

To validate the consistency of spatial labeling, spheroids were labeled by a combination of two dyes: Vybrant-DiL - a lipophilic dye which selectively stains the outermost layers of cells - and Calcein-AM - a hydrophilic dye which diffuses deep into spheroids (Fig. 2a, Fig. S1a). Quantifying dye intensity by flow cytometry revealed consistent labeling by Vybrant-DiL and Calcein-AM (Fig. 2b). Vybrant-DiL + cells are Calcein-AM high, whereas Vybrant-DiL- cells are intermediate or low on Calcein-AM and no cells are Vybrant-DiL- and Calcein-AM+. This pattern is consistent with the selectivity of Vybrant-DiL for the outermost layers of spheroids.

The radial position *r* of each cell was determined by scaling the log intensity of the Calcein-AM dye, to assign cells a radial position of *r* = 0 at the core, *r* = 1 at the periphery, and intermediate positions to cells in between. The number of cells increased quadratically with the radial position *r*, as expected from the geometry of spheres, Fig. S1b).

Taken together, these observations suggest that Calcein-AM intensities position cells along the core-periphery axis of spheroids.

Following dye quantification by flow cytometry, single cells were sorted into single wells and their transcriptome profiled by Smart-seq3xpress^28^, to associate the transcriptome of single cells to their radial position. Of 1,896 isolated cells, 1,527 (81%) passed flow cytometry and RNA sequencing quality control filters and were kept for further analysis (Methods, Fig. S1c). We call this approach Diffusion Smart-seq3 (in short: Smart-seq3D).

Smart-seq3xpress allowed deep characterization of the transcriptome, with a median 129,985 RNA molecules (Unique Molecule Identifiers - UMIs) identified per cell from 27,257 polyA + coding and non-coding human genes (Methods). Of these genes, we asked what fraction of genes had at least one UMI per cell on average, so as to facilitate detecting spatial regulation. 34% of the 27,257 polyA + genes had at least 1 UMI per cell at this sequencing depth (Fig. 2c, Fig. S1d). In comparison, sequencing by emulsion-based methods typically yield 5,000–25,000 UMIs per cell^30^. From these, one expects 3–14% of genes to have at least 1 UMI per cell (Fig. 2c, Fig. S1d). Thus, deep transcriptomics by Smart-seq3xpress could facilitate detecting spatial regulation without the need for computational methods that infer well-resolved transcriptional states by borrowing information across shallowly sequenced cells^31^.

### A statistical model overcomes gene expression noise to identify thousands of spatially-driven genes

To reveal associations between gene expression and radial position, one challenge is that spatial expression gradients are blurred by the stochasticity of transcription and mRNA decay, as well as by the sampling noise of single-cell transcriptomics^32–34^. For example, scattering the number of UMIs per cell as a function of the radial position for *VIM* and *VMP1* suggests more expression at the periphery and in the core, respectively (Fig. 2d-e). Yet, the number of UMIs varies strongly from cell to cell, even between cells with comparable radial positions. To reveal how a cell’s radial position regulates gene expression, gene expression and sampling noise needs to be separated from the signal of spatial regulation.

To do so, we introduce a probabilistic model to infer the spatial regulation function *f*_*g*_*(r)* of a given gene *g* from the noisy spatial UMI count data. *f*_*g*_*(r)* represents the fractional abundance of gene *g* in the transcriptome in cells at radial position *r*. For example, if mRNAs from a given gene represent 1 in 1000 mRNAs in cells with radial position *r*, *f*(r) = 1/1000. *f(r)* is unknown and is observed only through noisy measurements - the spatial UMI counts. Spatial UMI counts are thus modeled as noisy measurement of gene expression that depends on (1) the gene’s fractional abundance at that position *f(r)*, (2) the depth of sequencing in that cell, and (3) noise parameters specific to the gene (Methods).

We hypothesize that the dependency of gene regulation to space follows the parametric form of a second-order polynomial *f(r) = a + b r + c r*^2^. Here, *a* represents gene expression in the core (*r* = 0). Varying *b* and *c* respectively allows describing increasing and decreasing lines, upwards and downwards parabolae, and mixtures thereof. Spatial gene expression function whose linear *b* trend or quadratic trend *c* significantly differs from 0 represent spatially-regulated genes. We relax the assumption of a parametric form of spatial gene regulation later (see next section). By best-fitting *a*,* b*,* c* and noise parameters to the spatial UMI counts, we infer the spatial gene regulation *f(r)* of each gene.

The model faithfully captures the spatial expression of 9326 genes out of 9523 genes with at least one UMI per cell on average (Methods, log likelihood > -7500, Fig. S2a). For example, from the spatial UMI counts of *VIM*, the model infers that gene expression increases from the core to the periphery (Fig. 2d).

To validate this inferred spatial gene regulation function, we ask whether it is consistent with the spatial UMI counts measured by Smart-seq3D. Simulating spatial UMI counts from this spatial regulation function, we found that the distribution of simulated spatial UMI counts faithfully fits the distribution of spatial UMI counts of the Smart-seq3D data, including range and variability in expression between cells with similar radial positions (Fig. 2d). That the inferred spatial regulation is consistent with the measured spatial UMI counts supports the ability of the model to infer spatial gene regulation from Smart-seq3D spatial UMI counts. These observations generalize to *VMP1* — which is mainly expressed in the spheroid core (Fig. 2e) — and other genes (Fig. S2a).

Expression of 2363 out of 9325 genes (25%) significantly associated with space (false discovery rate fdr < 10% and regulation > 30%, Methods).

To investigate whether sequencing more RNAs could identify more spatial genes, we performed a downsampling experiment. The number of identified spatial genes grows with the number of UMIs per cell following a saturating sigmoid shape (Fig. S2b). This suggests that deeper transcriptome profiling is – on its own – unlikely to identify many more spatial genes.

To validate the spatial genes identified by Smart-seq3D, we performed immunofluorescence stainings. Smart-seq3D identifies *EGR1*, *FOS* and *JUN* as peripheral genes (Fig. S3a-c), consistent with the immunofluorescence staining patterns and with transcriptional activation of *EGR1* by the AP1 complex formed by the FOS-JUN dimer^35–37^. Immunofluorescence staining in the same spheroid model previously localized *VIM* and *Ki67* at the periphery^29^, consistent with the spatial gene regulation inferred by Smart-seq3D (Fig. S3d-e).

Smart-seq3D also finds *VMP1* to be expressed in the spheroid core, which matches the immunofluorescence spatial pattern (Fig. S3f).

### Diffusion Smart-seq3D reveals genes and pathways with diverse spatial expression pattern

**Fig. 3:**
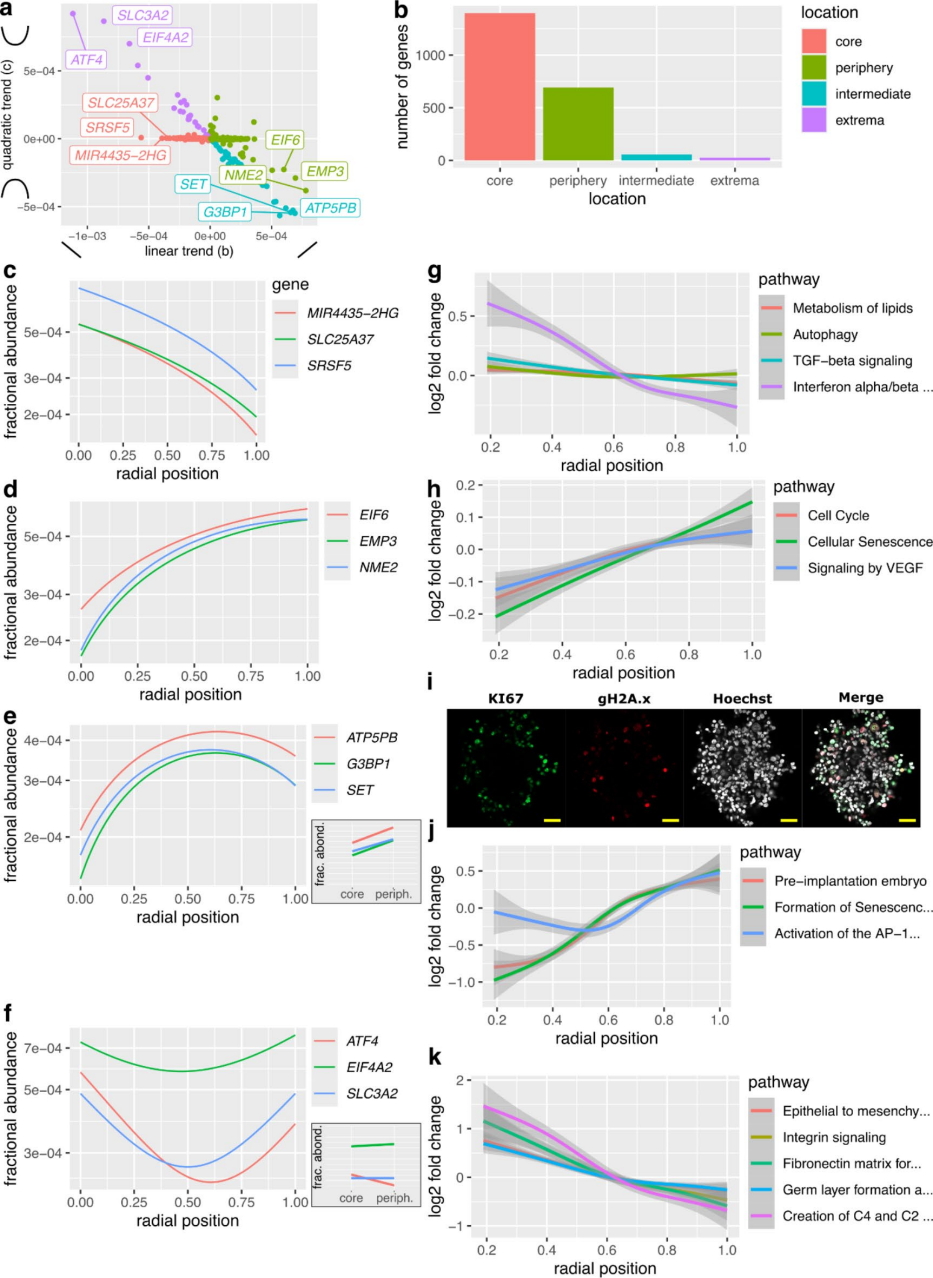
Smart-seq3D identifies thousands of spatial genes and assigns specific pathways to specific regions of tumor spheroids. **(a)** Smart-seq3D identifies genes with four spatial regulation patterns: core, periphery, intermediate and extrema. **(b)** Most genes are expressed in the core or in the periphery whereas genes expressed in the intermediate region or at extremes of the core-periphery axis are rare. **c. ***MIR4435-2HG*,* SLC25A37* and *SRSF5* are preferentially expressed in the spheroid core. The logarithmic scale allows representing genes with RNAs of different abundances on the same figure. **d.** *EIF6*, *EMP3* and *NME2* are upregulated in the spheroid periphery. **e.** Expression of *ATP5PB*, *G3BP1* and *SET* peaks in the intermediate region of spheroids. Inset: binning cells in two positional bins cannot reveal this spatial regulation pattern. Y-axis: same as in main figure. **f.** *ATF4*, *EIF4A2* and *SLC3A2* expression is lowest in the intermediate region and highest in the core and the periphery of spheroids. Inset: binning cells in two positional bins cannot reveal this spatial regulation pattern. Y-axis: same as in main figure. **g.** Genes expressed in the spheroid core are involved in lipid metabolism, autophagy, TGFb signaling and type I interferon signaling. **h.** Genes expressed in the periphery are involved in the cell cycle, cellular senescence and signaling by VEGF. **i.** Immunofluorescence imaging validates that the *KI67* proliferation marker and the γH2A.x marker of double-stranded breaks localize at the spheroid periphery. Scale bar: 100 μm. **j-k.** Non-parametric spatial pathway expression analysis. **j.** Genes involved in pre-implantation embryo, formation of senescence-associated heterochromatin foci and activation of the AP-1 complex are upregulated at the spheroid periphery. **k.** Genes involved in epithelial to mesenchymal transition, integrin signaling, fibronectin matrix formation, germ layer formation and complement activation are preferentially expressed in the core.

Examining the linear and quadratic parameters of the spatial expression functions inferred for the different genes of our dataset classifies genes into four patterns: genes expressed mainly in the spheroid core, genes expressed mainly in the periphery, genes expressed in the intermediate region half-way in between the core and the periphery, and genes expressed at extrema of the core-periphery axis but not in in the intermediate region (Fig. 3a).

Genes expressed in the core were most numerous (1398 genes, Fig. 3b), for example *MIR4435-2HG*,* SLC25A37 and SRSF5* (Fig. 3c). 691 genes - including *EIF6*,* EMP3 and NME2* (Fig. 3d) - were expressed in the periphery. 56 genes - such as *ATP5PB*, *G3BP1* and *SET* (Fig. 3e) were expressed in the intermediate region. Finally, 26 genes were expressed at the extremes of the core-periphery axis, among which *ATF4*, *EIF4A2* and *SLC3A2* (Fig. 3f). 192 genes had a spatial regulation pattern that did not clearly fit within these four patterns (Methods).

Thus, Smart-seq3D identifies genes with diverse spatial patterns. Two of these patterns - expression in the intermediate and at the extremes of the core-periphery axis - cannot be identified by a dual binning approach which sorts cells into two positional bins followed by single-cell gene expression profiling (Fig. 3e-f).

The diversity of spatial gene expression patterns identified by Smart-seq3D provides an opportunity to characterize the micro-environmental biology of TNBC spheroids.

Pathway enrichment analysis (Methods) suggests that cells at the core of the spheroid upregulate genes involved in interferon signaling, TGFb signaling, lipid metabolism and autophagy (Fig. 3g, Table S1, q < 10%).

In contrast, genes expressed by cells at the periphery of spheroids are involved in the cell cycle (Fig. 3h, Table S2). Possibly as a consequence of proliferation, cells at the periphery express genes involved in homology directed repair (fdr = 0.2%) of double-stranded DNA breaks (fdr = 2%, Table S2). Expression of cell cycle genes and double-stranded DNA breaks at the periphery is consistent with the experimental observation that the *KI67* proliferation marker and the γH2A.x marker of double-stranded breaks localize at the spheroid periphery (Fig. 3i).

The spheroid periphery also expresses senescence-associated genes and genes involved in signaling by VEGF (Fig. 3g, Table S2, fdr < 10%). High expression of genes expressed at the spheroid periphery associates with longer survival in the TCGA cohort (*p* < 0.01, Fig. S4a)^38^.

Genes expressed at intermediate positions between the core and the periphery or at the extremes of the core-periphery axis - that is lowest in intermediate regions - did not associate with known pathways (fdr < 10%).

To further characterize spatial gene regulation within spheroids, we relax the hypothesis that gene regulation follows a second-order polynomial dependency on radial position. We estimate the regulation of 6000 + pathways from Reactome^39^, KEGG^40^, Corum^41^ and WikiPathway^42^ by summing up the UMIs from genes involved in these pathways in each cell. Summing up UMIs from multiple genes decreases expression noise through the law of large numbers, thus alleviating the need for probabilistic inference of spatial regulation. We then test for association between pathway expression and radial position using the Xi correlation, a non-linear metric of statistical association (Methods)^43^.

The expression of 309 pathways (Table S3) significantly associates with radial position (fdr < 10%). On the periphery, genes associated with activation of the AP-1 family of transcription factors are upregulated (Fig. 3j). This is consistent with localization FOS and JUN — which dimerize to form AP-1 — at the spheroid periphery in immunostains (Fig. S3a-c). Genes involved in pre-implantation embryos and the formation of Senescence-Associated Heterochromatin Foci are also upregulated at the periphery (Fig. 3j).

On the other hand, genes upregulated in the spheroid core are associated with germ layer formation, epithelial to mesenchymal transition, integrin signaling, fibronectin matrix formation and the creation of the C4 and C2 complement activators. (Fig. 3k, fdr < 10%, Table S3).

Thus, depending on their location on the core-periphery axis, cancer cells from 3D spheroids express a diversity of transcriptional programs associated with embryonic states (pre-implantation at the periphery, post-gastrulation in the core), metastasis (EMT in the core), immunomodulation (complement, TGFb, interferon at the core), and survival (lipid metabolism, autophagy at the core).

### Spatial gene expression profiling of tumor spheroids by Smart-seq3D allows testing evolutionary principles of tumor heterogeneity

Profiling spatial single-cell gene expression in the simple, well-controlled system that are tumor spheroids can serve to test evolutionary principles of spatial tumor heterogeneity, such as multi-task evolution.

Multi-task evolution is a theoretical framework to interpret the diversity of traits of cells and organisms in terms of evolutionary trade-offs. The theory has found application from zoology to microbiology and paleontology to behavioral science^44–46^.

**Fig. 4:**
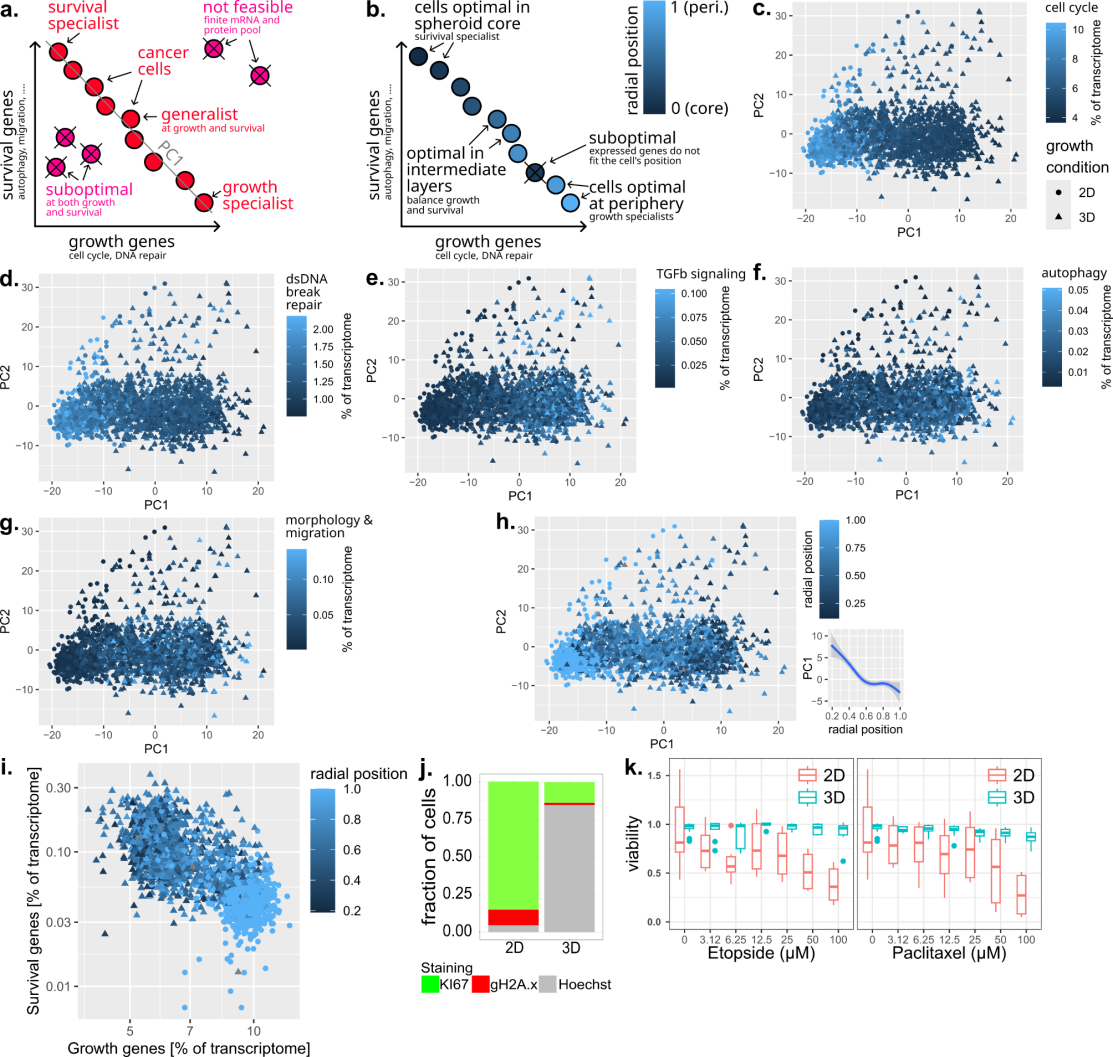
Performing Smart-seq3D on tumor spheroids allows testing evolutionary principles of spatial heterogeneity. **a-b.** Multi-task evolution theory makes two predictions regarding spatial tumor single-cell gene expression. **(a)** Prediction 1: when cells are cultured in a mix of two environments that require different genes, optimal transcriptomes are found on a linear gene expression segment. **(b)** Prediction 2: gene expression depends monotonically on radial position so that genes expressed optimally match the cells’ environment. **c-h.** Principal components analysis of gene expression of single cancer cells grown as 3D spheroids (triangles) suggests a 1D continuum of gene expression. 2D grown cells (circles) fall at one extreme of this continuum. **c-d.** 3D-grown cells at one end of the 1D continuum and 2D-grown cells upregulate genes involved in the cell cycle and in double-stranded DNA repair relative to 3D spheroids. **e-g.** At the other end of the continuum, 3D spheroids upregulate genes involved in TGFb signaling, autophagy and morphology / migration genes. 2D grown cells do not express these genes. **h.** Gene expression associates monotonically with the radial positions of cells. **i.** Consistent with multi-task theory (panels a-b), the transcriptional heterogeneity of cancer cells spans a continuum that trade-offs expression of growth genes vs. survival genes. The balance between the two associates with the radial position of cells along the core-periphery axis of spheroids. Growth genes from panels c-d, survival genes from panels e-g. **j**. The proportion of cells positive for the *KI67* proliferation marker and γH2A.x DNA damage marker is higher in 2D-grown cells than in 3D-grown cells. **k**. Cells grown in 2D are more sensitive to anti-proliferative (paclitaxel) and DNA damage-inducing (etoposide) treatments than cells grown in 3D.

In cancer, we previously conjectured that multi-task evolution could explain the spatial heterogeneity in genes expressed by single cancer cells in terms of optimal trade-offs between conflicting tasks that cancer cells need to perform depending on their environment^45,47^. For example, cancer cells need to both grow and survive to thrive but these two tasks require expressing different genes (Fig. 4a). This leads to a trade-off situation. To address this trade-off optimally, cells need to perform a different task depending on their environment, for example growth in an environment rich in oxygen and nutrients, and survival in a hypoxic environment poor in nutrients (Fig. 4b).

The multi-task evolution hypothesis that environmental selection for conflicting tasks explains spatial transcriptional heterogeneity has two testable predictions about the transcriptional architecture of spheroids.

First, a mathematical theorem^45^ shows that if each spheroid environment requires cancer cells to perform specific tasks and express specific genes, selection will constraint gene expression to the geometry of polytopes — lines, triangles, tetrahedra. With two environments for example, single-cell gene expression is predicted to be arranged as a 1D continuum – a linear segment (Fig. 4a). Cells at the end of the segment are specialists in an environment, whereas cells found in the middle of the segment are generalists that can thrive in intermediate environments — for example partial nutrient and oxygen depletion in intermediate spheroid layers. Cells off the segment are suboptimal and are discarded by evolutionary selection.

To test this prediction, we projected the transcriptome of 3D spheroid cells on their first principal components. Doing so reveals that transcriptional heterogeneity is arranged as a one-dimensional continuum (Fig. 4c-h), with the first principal component capturing 1.9x more variance than the second principal component (Fig. S5a). This observation suggests two environments in spheroids that require cancer cells to perform two conflicting tasks.

To characterize these tasks, we searched for pathways whose expression associates with the position of cells along the observed 1D continuum. At one end of this continuum, cells upregulate genes involved in the cell cycle (Fig. 4c) and double-stranded DNA break repair (Fig. 4d). Conversely, *TGFb*, autophagy and morphology/migration genes are upregulated at the other end of the continuum (Fig. 4e-g). This observation is consistent with cancer cells facing a trade-off between growth and survival.

A second prediction of multi-task evolution is that the position of cells along the 1D gene expression continuum associates monotonically with the cell’s radial position, so that gene expression optimally matches the cell’s environment (Fig. 4b). Consistent with this prediction, the radial position of cells correlates with their position along the 1D gene expression continuum (Fig. 4h).

To match tasks to spheroid environments, we sum up the expression of growth and survival genes from the pathways highlighted in Fig. 4c-g and associate their expression to the radial position of cancer cells within the spheroid (Fig. 4i). We find that cells at the periphery of the spheroid express growth genes whereas cells at the core express survival genes (Fig. 4i), consistent with selection for growth at the periphery and selection for survival in the spheroid core.

To test the hypothesis that the richer, less crowded peripheral spheroid environment favors growth, cancer cells were grown in 2D culture — a nutrient- and oxygen-rich environment with low cellular crowding — prior to performing single-cell transcriptomics. Cells grown in 2D fell close to cells from the spheroid periphery characterized by high proliferation and double-stranded DNA breaks on the one-dimensional gene expression continuum (Fig. 4c-i, Fig. S5b).

The observation that a richer environment selects for growth is further supported by immunostainings: the proportion of proliferative *KI67* + cells increased from 16 ± 10% in 3D to 85 ± 5% in 2D (Fig. 4j). Immunostaining for the γH2A.x DNA damage marker, we find 10.5% of positive cells in 2D vs. 1.77% in 3D (Fig. 4j).

That a richer, less crowded environment selects for the task of growth implies that cells in that environment should be more sensitive to anti-proliferative treatments as well as genotoxic treatments due to their higher basal level of proliferation-induced DNA damage. Treatment with the tubulin assembly inhibitor Paclitaxel or the DNA damage inducing drug Etoposide indeed reduced the viability of 2D-grown cells with dose-dependent toxicity whereas the spheroid showed resistance (Fig. 4k).

Multi-task evolution explains two other observations from the Smart-seq3D data.

If there are only two environments in tumor spheroids, multi-task evolution predicts that few genes should be specifically expressed in intermediate layers because there is no specific task to be performed by specific genes there. Instead, balancing expression of growth and survival genes — a generalist gene expression profile — is predicted to be optimal in intermediate layers. This is consistent with the observation that few spatial genes are expressed specifically in intermediate spheroid layers (Fig. 3b).

Similarly, the observation that few genes are expressed specifically at the extremes of the core-periphery axis (Fig. 3b) is consistent with multi-task evolution. For a gene to be expressed at the extremes of the core-periphery axis, it needs to benefit cancer cells in both the core and the peripheral environments. If there are only two environments however, this gene would be beneficial in all environments. Therefore, it needs to be expressed ubiquitously throughout the spheroid. Because of this, there is no selection pressure for genes specifically expressed at both extremes of the core-periphery axis.

Thus, Smart-seq3D in tumor spheroids allows testing evolutionary principles about the spatial transcriptional heterogeneity of single cancer cells.

### Smart-seq3D reveals aspects of transcriptional heterogeneity of *in vivo* tumors captured by 3D spheroids or missing from them

The observation that the transcriptional heterogeneity of 3D spheroids is not modeled by 2D cultures and that 3D spheroids express spatial genes similar to genes associated with inter-tumor diversity could position 3D spheroids a step closer to tumors. Alternatively, the transcriptional heterogeneity of 3D spheroids may be irrelevant to tumors *in vivo* because cancer cells *in vivo* may be heterogeneous in different ways than 3D spheroids.

**Fig. 5:**
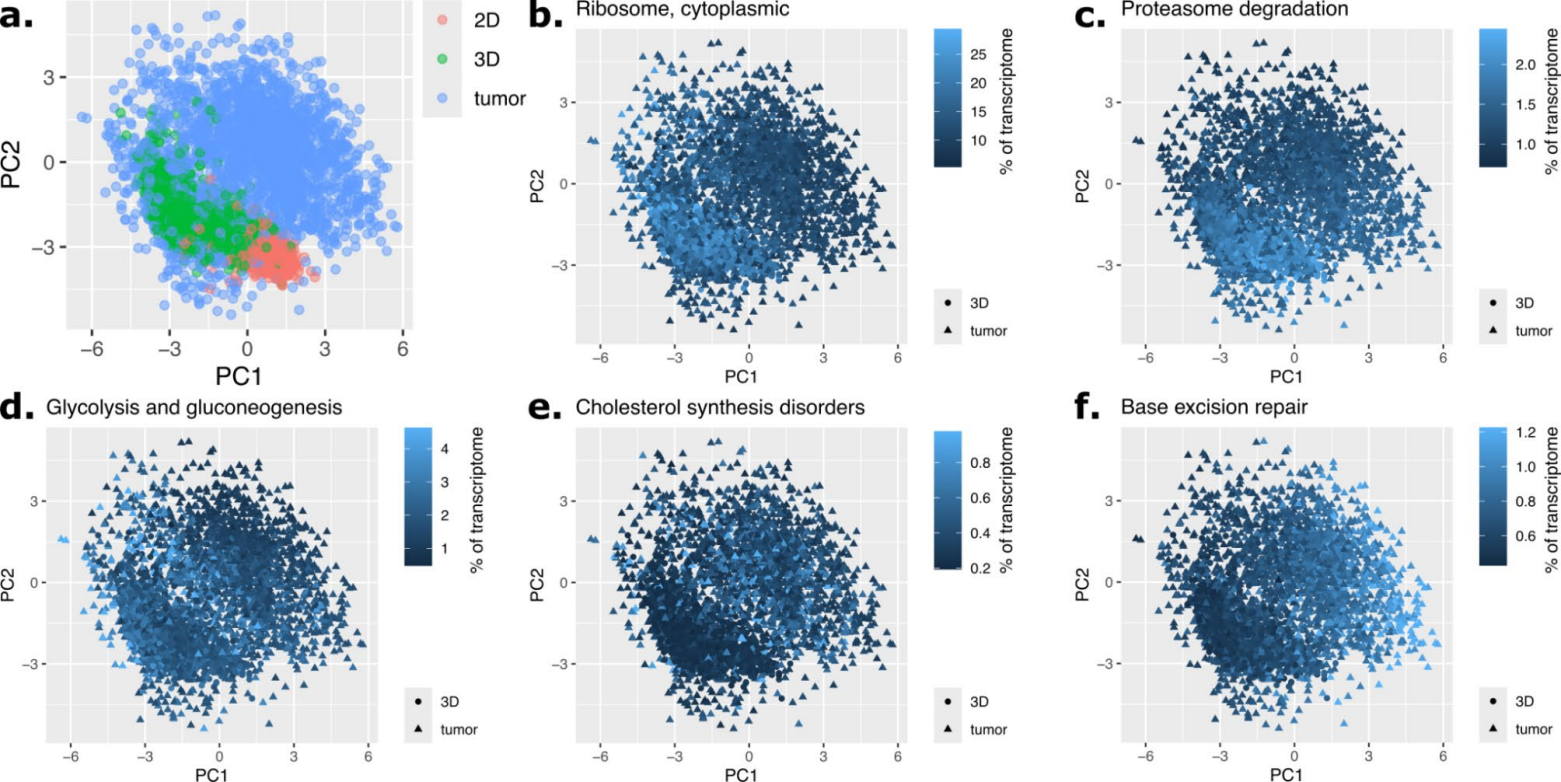
Smart-seq3D reveals aspects of transcriptional heterogeneity of *in vivo* tumors captured by 3D spheroids or missing from them. **a.** 3D spheroids mimic a larger portion of the transcriptional heterogeneity of tumors than cells grown in 2D. Principal components analysis of single cancer cells gene expression from a triple-negative breast tumor. Cells grown in 2D and 3D are projected on the same space. Data: Wu *et al*. **b-d.** 3D spheroids capture transcriptional states of *in vivo* cancer cells characterized by high expression of ribosomal genes (b), proteosome genes (c) and genes involved in glycolysis and gluconeogenesis (d). **e-f.** 2D cultures and 3D spheroids do not recapitulate transcriptional states of *in vivo* cancer cells characterized by high expression of genes involved in cholesterol synthesis disorders (e) and base excision repair (f).

We tested this using the Smart-seq3D data. Cells cultured in 2D and 3D were projected onto the first two principal components of 2279 cancer cells from a triple-negative breast tumor (Fig. 5a, data:^30^). Cells from 2D and 3D cultures overlapped with cells from tumors. This suggests that 2D and 3D cultures model some of the transcriptional heterogeneity of tumors.

Compared to cells grown in 2D, cells from 3D spheroids recapitulated a larger extent of the transcriptional heterogeneity of *in vivo* cancer cells (Fig. 5a). In particular, pathway analysis of genes differentially expressed *in vivo* compared to 3D spheroids showed that 3D spheroids successfully recapitulated *in vivo* transcriptional states characterized by high expression of genes involved in the ribosome, in the proteosome and in glycolysis/gluconeogenesis (fdr < 10%, Fig. 5b-d, Table S4, Methods).

On the other hand, a large fraction of cancer cells *in vivo* expressed genes involved in cholesterol synthesis disorders and genes involved in base excision repair higher than observed in 3D spheroids (fdr < 10%, Fig. 5e-f, Table S4). Optimizing 3D spheroid growth conditions or genetically engineering subclonal cellular populations that upregulate these genes could thus deliver *in vitro* models more faithful to *in vivo* tumors.

## Discussion

Spatial transcriptomics can synergize with 3D culture methods to illuminate tissue biology and serve as a platform for drug development but there are technical and financial obstacles. To address these, we introduce Smart-seq3D. Cells grown as 3D spheroids are stained with dyes of increasing intensity along the core-periphery axis, followed by deep single-cell RNA sequencing by the Smart-seq3xpress protocol, to produce deep spatial UMI counts. From these deep spatial UMI counts, a probabilistic model infers the spatial regulation function of each gene to identify 2000 + spatial genes, 10x more than expected at the sequencing depth of typical emulsion-based single-cell RNA sequencing experiments. Genes and pathways are identified that have spatial regulation patterns that cannot be seen by two-bin sort-and-sequence approaches. Smart-seq3D can be used to test evolutionary principles of the spatial transcriptional heterogeneity of tumors and to explore aspects of the transcriptional heterogeneity of *in vivo* tumors captured or missed by 3D spheroids or 2D cultures.

One potential caveat of Smart-seq3D is that the Calcein-AM has been reported to be efluxed out of some cancer cells^48^, thus preventing spatial positioning. While calcein-AM eflux was not an issue in our experiments, it could be replaced by Hoechst 33,342 if necessary. This substitution however requires access to a UV laser. Another limitation of the method in its current setting is that labeling of cancer cells with antibodies can be challenging due to the enzymatic dissociation step by trypsin which affects the extracellular matrix and surface antigens. To address this, optimization can identify trypsin resistant epitopes or substitute trypsin with other enzymes. Mechanical dissociation was evaluated during the development of the method but was associated with significant cell death during the process.

The present study employs dye diffusion as a measure of a cell’s position on the core-periphery axis. This is appropriate in the context of 3D cell cultures with near-spherical geometry because the amount of dye taken up by a cell depends on the distance to the surface of the 3D culture, which is measured by the radial position in a spherical 3D culture. In non-spherical 3D cultures, dye diffusion can still be used to position cells with respect to the culture’s surface, with the caveat that areas with high curvature create local diffusion patterns that differ from flatter regions^49^. Future experiments could explore dye diffusion in non-spherical 3D cultures by combining clearing agents with multiphoton microscopy, as this technology can overcome imaging depth limitations of confocal microscopy. Multiphoton microscopy allows measuring fluorescence signals 2 to 10 times deeper than classical laser scanning confocal microscopes accessible in most institutions^50,51^. Beyond this caveat, Smart-seq3D requires appropriate spheroid compactness: loose 3D cultures in which the dye diffuses too easily do not establish clear gradients.

Another caveat is that the inferred spatial gene regulation patterns could be confounded by intrinsic cell states. For example, if cells spontaneously transition in and out of the cell cycle and if cycling causes cells to relocalize to the spheroid periphery, we expect correlation between the spatial location and expression of many genes dependent on the cell cycle, even in the absence of direct spatial regulation of these genes.

We tested this hypothesis by multivariate regression of gene expression on the core-periphery position of cells and their intrinsic state in terms of cell cycle and stress genes. We found that controlling for cell cycle and stress has little impact on spatial - gene expression associations (Fig. S6a-b). Spatial gene regulation was similar when directly associating expression to space compared to when controlling for cell cycle and stress (*r* = 0.93, Fig. S6a).

The main exceptions to this trend are a handful of ribosomal proteins and the ribosomal maturation gene *NPM1* which are specifically expressed at the periphery, an effect that is explained by their association to cell cycle and stress. Conversely, a handful of mitochondrial genes are specifically expressed at the core, an effect which is explained by their association to cell cycle and stress. We found no example of genes whose association to space increased after correcting for cell cycle and stress. This suggests that cell cycle and stress do not mask space-expression association in our spheroid system.

Beyond identifying spatial genes in cancer, tumor spheroids Smart-seq3D could serve as a well-controlled experimental system amenable to rapid experimentation and intervention to further develop and test evolutionary theories of spatial transcriptional heterogeneity^47,52,53^. For example, the one-dimensional expression gradient of genes and pathways along the periphery-core axis suggests optimal adaptation of cancer cells to a progressively nutrient deprived and crowded environment, from growth in peripheral layers to survival strategies such as autophagy and lipid metabolism in the spheroid core (Figs. 3 and 4^47^), . To test the optimal gene expression hypothesis, *in vitro* evolution experiments could monitor how this gradient is progressively established upon passaging cancer cells that are initially poor at forming spheroids, whether this gradient persists to passaging tumor spheroids, and if this gradient can be lost by passing spheroid-forming cancer cells in 2D for increasing periods of time. Genetic screens could explore key regulators of the grow-survival gradient and whether perturbing these regulators decreases cancer cells’ fitness.

An alternative and more mechanistic interpretation of the transcriptional heterogeneity of 3D spheroids is that cancer cells mis-interpret the spatial context of spheroids as different embryonic layers: the surface of pre-implantation embryo at the periphery, the forming germ layers at the core. This observation could thus represent an illustration of the notion that embryonic and developmental programs are hijacked in malignancy^54^.

Several observations suggest that tumor spheroids have relevance to the biology of tumors. Aspects of tumor transcriptional heterogeneity not captured by 2D cultures are captured by spheroids (Fig. 5). The growth and survival tasks of cancer cell in spheroids associate with pathways previously identified to support two out of five universal cancer tasks by tumor genomics studies^55^, namely the ‘cell division’ and ‘invasion and tissue-remodeling’ tasks. Peripheral gene signatures are associated with longer survival (Fig. S4a). This observation could stem from higher proliferation and dsDNA breaks at the periphery, which sensitizes cells to chemotherapy and genotoxic treatments. In addition, proliferating cells may be less prone to invade surrounding tissue or shed from the tumor to form distant metastases - the grow-or-go hypothesis^56^. Thus, evolutionary principles learned in spheroids could translate to human tumors. Targeting genes expressed in the spheroid core and periphery could guide the development of therapies that interfere with multiple cancer tasks to decrease resistance^29,47^.

The spheroid core expresses signals with immuno-suppressive potential such as Interferons and TGFb^57,58^. This could position 3D spheroids as an experimental system to develop drugs effective against heterogeneous tumors. Co-culturing 3D spheroids with cytotoxic lymphoid cells or other immune cells could serve as a screening platform for interventions aimed at countering immuno-suppressive signals of cancer cells. Diffusion smart-seq3D would be well suited for this task, because of its scalability, cost-efficiency (less than 1 USD per cell after initial investment in infrastructure - liquid dispenser, cell sorter, sequencer^28^ and ability to interrogate the deep transcriptome and thus discover new immunobiology.

In recent years, approaches have been developed to segment tissue into spatial domains by integrating transcriptomes (including cell type) and proximity information using deep learning^59^. Such approaches may have relevance in interpreting Smart-Seq3D data. In fact, the dedicated computational methodology introduced in this article bears conceptual similarities to deep-learning-based methods: both infer a latent variable (here gene-specific regulatory functions) by integrating spatial information (here the position of cells on the core-periphery axis).

Unlike deep-learning-based methods, our approach considers one gene at a time. While doing so potentially leaves information from gene-gene correlations unexploited, there are benefits in terms of (i) interpretability, by letting the gene-centered data speak for itself with less abstraction caused by layers of inference, and (ii) less risk to infer false-positive gene-gene correlations - a real concern when interpreting data of sparse nature such as single-cell transcriptomics^34^. In addition, specializing our approach for Smart-seq3D data - modeling 1D positional continua, modeling gene expression stochasticity - allows introducing probabilistic considerations to rigorously quantify the statistical evidence for spatial regulation. Doing so is challenging by deep learning.

Future methodological development could combine ideas from both approaches for optimal interpretation of spatial data.

Future work could also expand single-cell RNA sequencing to protocols for co-profiling of RNA and DNA^60^. 3D culture methods that induce aspects of *in vivo* transcriptional heterogeneity presently under-represented in 3D spheroids — respiratory electron transport, cholesterol biosynthesis, FOS-JUN / AP-1 — could be developed to establish *in vitro* models more faithful to tumors *in vivo*.

## Methods

### Reagents information

All reagents’ data are provided in the supplementary Table S5 including suppliers and dilution used when applicable.

### Spheroid culture

The MDA-MB-231 S was obtained from the Karolinska Institutet and was authenticated using EurofinsGenomic (D7S820: 8,8; CSF1PO: 13,13; TH01: 7,9,3; D13S317: 13,13; D16S539: 12,12; vWA: 15,15; TPOX: 9,9; AMEL: X,X; D5S818: 12,12) and tested for mycoplasma. The cells were maintained in DMEM, FBS 10%, Penicillin-Streptomycin 1% and Mycoexpert. All the cell lines were grown at 37 °C, 5% CO_2_ and 100% humidity. No experiment used cells maintained for more than 10 passages to reduce the risk of genetic drift. The cells were washed twice with PBS to remove the non-adherent or dead cells, trypsinized for 5 min at 37 °C and quenched with culture medium. The viability was estimated using a Cellcounter2 and 0.4% trypan blue solution according to manufacturer instructions. If the viability was below 90%, the cells were centrifuged at 500 g for 5 min and the pellet was resuspended in medium to remove the dead cells. For the spheroid culture ~ 5000 live cells were seeded on 1% agarose coated plates. Reproducible spheroids form in ~ 5 days for the MDA-MB-231 S.

### Dye diffusion

The dye diffusion to spatial profiling of tumor spheroids has been extensively applied using Hoechst 33,342 and flow cytometry for functional investigation of cellular activities^22,24^ and recently adapted to single-cell transcriptomics^23,25^.

We used here the Vybrant-DiL and Calcein-AM dyes: depending on the available flow cytometer, these dyes can be replaced by Hoechst 33,342 or other lipophilic dyes^23,24^.

To evaluate the dyes diffusion, the spheroid previously incubated with Vybrant-DiL and Calcein-AM were transferred to 1.5mL tubes, washed twice with 1mL of PBS and centrifuged at 300 g for 5 min. The pellet (~ 20 spheroids) was then resuspended in 150µL of PBS and 150µL of 4% paraformaldehyde and incubated for 60 min at room temperature, the fixation is then stopped by the addition of Glycine 0.1 M in PBS solution. The quenched fixative is removed and the spheroids were washed twice in PBS-Tween20 0.2% Glycine 0.1 M. The spheroids were then incubated in PBS-Tween20 0.2% Glycine 0.1 M containing Hochest 33,342. Following the staining the spheroids were washed twice in PBS-Tween20 0.2% and once in PBS. The liquid is then replaced by 80µL of the clearing agent Omnipaque. The cleared structures were then transferred into a silicon gasket covered with glass coverslips prior to imaging with the LSM980-AiryScan2 (Zeiss). A representative image of the dye diffusion is in Fig. 2a.

### Flow cytometry

The spheroids were incubated for 2 h with 10µM of Vybrant-DiL (or DiL) and Calcein-AM. The gradient of staining occurs from the outer to the inner layer of the spheroids as illustrated in Fig. 2a. Longer term incubation (14 + days) of the cells with the tested dyes was not associated with cellular toxicity (90–95% viability using trypan blue assay on dissociated spheroids). Following observation under an inverted microscope, all the spheroids from a 96-well plate that kept spherical structures were transferred to 15mL tubes, washed twice with 10mL of PBS and centrifuged at 300 g for 5 min. The pellet (~ 60 spheroids) was then digested by the addition of 200µL of trypsinLE for 5 min and mixed by pipetting ~ 10 times prior to the addition of 100µL of culture medium. The cells were then washed twice in PBS, and filtered through a 35 μm nylon mesh capped tube. One drop of propidium iodide was added to the cell suspension to filter out dead cells during sorting (Figure S1a, Comp-PI* Hi cells). Cellular debris with low size (FSC-A Low) and granularity (SSC-A Low) were discarded (Figure S1a). Individual cells (based on FSC-W) and granularity (SSC-A) compatible with live cells were sorted in 384-well plates with a Melody flow cytometer and the features of the sorted cells were recorded: SSC, FSC, FITC (Calcein-AM), PE.Cy5 (Vybrant-DiL).

### Sequencing and data processing

2602 single cells were sorted in 384-well plates, 1896 cells from spheroids and 768 from adherent cells (Fig. S1a, c), and processed identically to the Smart-seq3xpress protocol extensively detailed in Hagemann-Jensen *et al*.^28^. zUMIs version 2.9.7 was used to process raw FASTQ files^61^. Reads were mapped to the human genome (hg38) using STAR version 2.7.3^62^, which identified 9523 genes among all cells (at least one UMI in any cell).

### Immunostaining

Adherent cells or spheroids were washed twice in PBS and fixed with 4% paraformaldehyde. The fixation was followed by a quenching step with Glycine 0.1 M in PBS and two washes with PBS-Tween20 0.2%. A blocking step with 5% goat serum, PBS-Tween20 0.2% Glycine 0.1 M was performed prior to overnight incubation with primary antibodies in the same buffer. The next day the cells were washed twice with PBS-Tween20 0.2% and stained overnight with the secondary antibodies and Hoechst 33,342 in the blocking solution. The next day the stained cells were washed twice in PBS-Tween20 0.2% and one wash with PBS. The adherent cells were observed directly using an AxioObserver (Zeiss) equipped with Colibri7 (Zeiss) and Hamamatsu C11440 (Hamamatsu) camera while the spheroids were cleared for 2 days in Omnipaque. The cleared structures were then transferred into a silicon gasket covered with glass coverslips prior to imaging with the LSM980-AiryScan2 (Zeiss). Confocal images were acquired every 10 μm (Settings, 1AU for each fluorochrome) through the spheroids. All the images were converted from .czi to 8bit .tiff using ZenBlue software and exported using the ‘original data’ and the ‘full set of dimensions’ settings. The exported images were analyzed using CellProfiler4 ^63^. The results were then analyzed in R/RStudio.

### Live/dead staining

Following the different treatments, cell death was assessed directly in the 96 well plates by adding 0.5µL/mL of propidium iodide and Hoechst 33,342 both at 1 mg/mL in water. After 1 h of incubation at 37 °C, 5% CO_2_, images from each well were acquired at 5x magnification using a Zeiss AxioObserver equipped with a Hamamatsu camera system. Bright field, λ_ex/em_ = 405/461nm, λ_ex/em_ = 538/619nm were sequentially acquired in continuous mode with Zen Blue. The positive control for cell death used 0.1% Triton-X100 treatment for 30 min. The viability was measured as the area positive for propidium iodide divided by the area positive for Hoechst 33,342.

### Probabilistic inference of spatial gene regulation from spatial UMI counts

We describe an analytical approach to identify spatially regulated genes from Smart-seq3D data without (i) clustering single cells according to genes expressed nor (ii) discretizing cells into spatial bins. This has benefits of sensitivity.

(i) To reveal associations between gene expression and radial position, two methodological approaches are possible.

One approach first groups single cells into clusters of similar transcriptome, and then tests whether different clusters have different positions among the core-periphery axis. This has the benefit of being practical, through following the standard single-cell RNAseq analysis pipeline^64^.

A second approach examines genes one by one, testing for association between gene expression and radial position. This has the benefit of sensitivity. To see why, note that transcriptional heterogeneity can be driven by both space-dependent and space-independent factors. Space-independent factors can mask space-dependent factors by clustering cells, through averaging expression of spatial genes across cells of different spatial locations. To illustrate this, we consider the limiting case of a space-independent factor - the cell cycle for example - dominates the impact of a spatial factor - hypoxia for example - on the transcriptome. In this instance, cells will cluster according to cell cycle phase with no spatial association, masking the effect of hypoxia. To avoid this, we follow an approach to identify individual genes whose expression associates with radial position.

(ii) As the analysis’ first step, the radial position of cells could be gated into bins of cells with similar radial position, as is commonly done with flow cytometry data. Yet, gating radial positions requires defining an arbitrary number of gates along with their thresholds and can limit the diversity of spatial patterns that can be observed. To see why, consider the extreme case in which cells are gated into core and peripheral bins: such a gating scheme cannot discover genes and pathways whose expression peaks half-way between the core and periphery of spheroids. Adding an intermediate bin can address this specific issue but with limitations in identifying subtle spatial patterns. To maximize sensitivity, we thus analyze radial positions as a continuum without gating.

Our analysis hypothesizes that the dependency of gene regulation to space follows the parametric form of a second-order polynomial *f(r) = a + b r + c r*^*2*^. This presents advantages in terms of simplicity (just 3 parameters), parameter interpretation (a is the expression at the core, b and c are the linear and quadratic trends), and generality (capturing non-linear and non-monotonic functions). Adding the 2^nd^ order term provides a better approximation to the true underlying spatial gene regulation function than a 1^st^ order polynomial, and there are theoretical bounds on the error of the approximation as per Taylor’s theorem. The mathematical details of the probabilistic inference model of spatial genes from Smart-seq3D data are provided in the Supplementary Methods (Supplementary Material 1).

Instead of a second order polynomial, higher-order polynomials or generalized additive splines could be used to reveal more complex spatial regulation functions. We did not explore these for three reasons.

First, the data suggests that more complex spatial regulation functions are unlikely in this system. For example, fitting a second order polynomial spatial regulation function shows that the expression of most genes peaks in the core or at the periphery whereas genes whose expression is non-monotonic - with a maximum or minimum at intermediate radial position - are rare (Fig. 3a-b). This is consistent with a core-peripheral environmental gradient in our spheroids. Non-parametric smoothing of the spatial gene expression suggests spatial gene regulation functions that can be captured by second order polynomials (compare Fig. 3j-k and c-f).

Second, more complex spatial regulation functions would be difficult to validate from the gene expression data due to stochasticity in single-cell gene expression and profiling (see Fig. 2d and e). Immunofluorescence microscopy can distinguish genes expressed at the core or at the periphery, but more complex patterns are difficult to validate (Fig. S3). If more complex spatial regulation functions cannot be validated, there is limited value in trying to identify them.

Third, there are non-trivial numerical optimization challenges, even with a simple 3-parameter polynomial and a gene-specific negative binomial size parameter. For example, fitting needs to ensure that parameters aren’t negative. Multi-dimensional optimization give parameter a (expression at the core) arbitrarily small values at which the likelihood function is flat, which leads to an ill-conditioned Hessian matrix and thus trouble when estimating error bars through a Gauss/Laplace approximation of the likelihood function. We addressed this using a weak prior on minimal gene expression given sequencing depth. In genes where the linear and quadratic trends are weak, the likelihood can be flat in the linear and quadratic trends b and c too, which we addressed using a weak ‘no trend’ prior. Convergence requires starting with a reasonable parameter set, for which we developed a heuristic that assumes weak spatial trend (for which the negative binomial size parameter can be estimated analytically) and no quadratic trend (which simplifies the parameter space and thus speeds up pre-optimization), before fitting the full model.

This suggests that extending the present framework to more complex spatial patterns would be a significant methodological endeavor, which we reserve to future studies.

Future studies could also explore other choices of priors. Here, we employed uninformed priors on the parameters of the spatial gene regulatory functions. Stronger, informative priors - using empirical distributions or hierarchical models that share variance across genes to stabilize parameter estimates for example - could have benefits of power and accuracy in inferring more complex spatial gene regulatory functions, particularly for low-abundance transcripts.

### Downsampling analysis

To examine how decreasing the depth of RNA sequencing affects the number of spatially regulated genes, we performed a down-sampling analysis. For each cell *c* and gene *g*, we sampled UMI counts *n’*_gc_ from a binomial distribution with success probability *p* and total number of trials *n*_*gc*_, the number of UMIs for gene *g* in cell *c* in the Smart-seq3D data. We successively set *p* to 0.3%, 1%, 3%, 10% and 30%, generating 5 downsampled Smart-seq3D UMI count matrices *n’*_*gc*_ for each value of *p*. From the *n’*_*gc*_ matrices, we inferred spatially regulated genes as described below.

### Pathway enrichment analysis

To identify pathways expressed in the core, periphery, intermediate layers and extrema of spheroids, we tested for enrichment of genes from known complexes and pathways at these different spheroid locations.

To do so, we collected genes expressed in a given location (core, periphery, intermediate or extrema) as defined by their expression pattern (Supplementary Material 1). Only genes with log-likelihood L>-7500, fdr < 10% and regulation > 30% were considered. Genes were ordered by magnitude of regulation across radial positions and passed to the gost() function of g:Profiler2 ^65^, as an ordered query to compute GSEA-style p-values based on a running sum statistic.

From p-value distributions, the fdr was estimated using the Benjamini-Hochberg procedure. The universe of genes was defined as all genes with at least one UMI per cell on average. Table S2 and S3, we report the resulting over-represented pathways at a fdr < 10%.

The same procedure was used to identify pathways whose expression associates with the position of cells along the first principal component of gene expression of 3D spheroids, by ordering genes according to their loading on the first principal component.

To identify pathways expressed *in vivo* but not in 3D spheroids, we computed the log-fold-change of gene expression *in vivo* compared to 3D spheroids, and then performed pathway enrichment on the resulting sorted gene list. Doing so carries the risk of identifying pathways involving genes that are upregulated in all *in vivo* cancer cells, potentially due to differences in gene expression profiling technology. To control for this, we also require that pathway expression increases *in vivo* from the region of gene expression space occupied by 3D spheroids (bottom left of panel 5a) to the region farthest away from it (top right of panel 5a). We achieved this by performing pathway enrichment analysis on a gene list ranked by the loading of each gene on PC1 + PC2. The intersection of pathways enriched in both tests represents pathways expressed *in vivo* but missing from 3D spheroids.

To identify pathways expressed both *in vivo* and in 3D spheroids, we similarly search for pathways upregulated in 3D spheroids compared to cells *in vivo* and whose expression increases along PC1 + PC2.

### Non-parametric identification of spatially associated pathway

To relax the assumption of a 2^nd^ order spatial regulation function, we estimated pathway expression by summing up, in each cell, the UMIs of genes from pathways (WikiPathways, Reactome) and known complexes (Corum) was summed up and normalized to the total number of UMIs in each cell to estimate the fractional abundance of RNAs from that complex/pathway within each cell’s transcriptome. Pathways with less than 5 genes in our dataset were not considered for further analysis.

We then tested for non-linear association between these and the radial positions of cells using the Xi correlation^43^(XICOR R package). From the distribution of p-values, we estimated the fdr using the Benjamini-Hochberg procedure. The positional expression of each complex/pathway along the core-periphery axis avgR was estimated by binning cells into 10 spatial bins, averaging the fractional abundance of each pathway/complex over the cells in each bin, and computing the average radial position of this pathway weighted by the fractional abundance in each bin.

## Electronic supplementary material

Below is the link to the electronic supplementary material.

Supplementary Material 1

Supplementary Material 2

Supplementary Material 3

Supplementary Material 4

Supplementary Material 5

Supplementary Material 6

Supplementary Material 7

## Data Availability

The data generated during the current study are on ArrayExpress accession number: E-MTAB-12325. All scripts can be found on our GitHub repository, link: https://github.com/jhausserlab/diffusionSmartSeq3.git.
