# Supplementary material for: Diffusion Smart-seq3 of breast cancer spheroids to explore spatial tumor biology and test evolutionary principles of tumor heterogeneity
Source: Sci Rep. 2025 Jan 30;15:3811. doi: 10.1038/s41598-024-83989-x (PMC11782488; doi:10.1038/s41598-024-83989-x)
Supplement: Supplementary file 1 — Supplementary Material 1 [file 41598_2024_83989_MOESM1_ESM.pdf]

1 Supplementary methods

1.1 Inferring spatial gene regulation from Smart-seq3D data

Smart-seq3D quantifies (i) the number of Unique Molecular Identifiers (UMIs) n_{gc} of gene g in the cell c, and (ii) the radial position $r_c \in [0,1]$ of that cell. From this, we wish to infer the spatial regulation functional $f_g(r)$ which indicates the fractional abundance of mRNAs from gene g in the transcriptome of cells found at position r of the spheroid.

The challenge in inferring f_g from n_{gc} is that n_{gc} is a stochastic quantity: it is related to f_g with random noise due to (i) stochasticity in gene expression and (ii) the single cell transcriptomics sampling process (here by Smart-seq3xpress) [1, 2, 3].

We address this using probabilistic inference [4]. Specifically, from the Smart-seq3D data n_{gc} and r_c, we seek to determine the likeliest spatial regulation function, parameterized as $f_g(r) = \alpha + \beta r + \gamma r^2$. Here, $\alpha = f_g(0)$ represents the fractional abundance of gene g in cells located in spheroid core. β is the linear spatial regulation trend. γ represents the quadratic spatial regulation trend. Note that, in this section, we use α, β, γ instead of a, b, c for the gene expression in the core a, the linear trend b and the quadratic trend c so as not to confuse the quadratic trend c with the cell c.

Using Bayes' theorem, the probability of the parameters α, β, γ given the Smart-seq3D data n_{gc}, r_c can be written as

$$P(\alpha, \beta, \gamma | n_{gc}, r_c) = \frac{P(n_{gc}, r_c | \alpha, \beta, \gamma) P(\alpha, \beta, \gamma)}{P(n_{gc}, r_c)}$$

Here, $P(n_{gc}, r_c)$ does not depend on the parameters α, β, γ. Because of this, inferring the likeliest parameters α, β, γ by maximizing the left side of the above equation can ignore the term $P(n_{gc}, r_c)$.

To define $P(n_{gc}, r_c | \alpha, \beta, \gamma)$, we initially explored a Poisson distribution

$$P(n_{gc}, r_c | \alpha, \beta, \gamma) = \frac{\mu^{n_{gc}} e^{-\mu}}{n_{gc}!}$$

with mean $\mu = f_g(r|\alpha, \beta, \gamma) N_c$ with $N_c = \sum_g n_{gc}$ the total number of UMIs in cell c, following previous work [3]. However, upon simulating genes from best-fitted distributions, we observed that modeling n_{gc} as a Poisson distribution underestimates the variance in gene expression. Following previous work on stochasticity in gene expression [1, 2], we thus model n_{gc} as a negative binomial distribution,

$$P(n_{gc}, r_c | \alpha, \beta, \gamma, \sigma) = \binom{n_{gc} + \frac{\mu^2}{\sigma^2 - \mu} - 1}{n_{gc}} \left(1 - \frac{\mu}{\sigma^2}\right)^{n_{gc}} \left(\frac{\mu}{\sigma^2}\right)^{\mu^2/(\sigma^2 - \mu)}$$

with σ the variance of gene g. In addition, we employ a flat, non-informative prior on α and Gaussian priors on β and γ,

$$\log P(\alpha, \beta, \gamma) = k - \frac{1}{2}\left(\frac{\beta}{10^{-3}}\right)^2 - \frac{1}{2}\left(\frac{\gamma}{10^{-3}}\right)^2$$

where k is a constant independent of α, β, γ. This prior assumes no spatial trend (mean 0) until we see the Smart-seq3D data and formalizes that most genes have spatial trends smaller than 10^{-3}. The reason for this is that mRNAs from genes with highest expression have fractional abundance less than 10^{-3} (Fig. 2a-b). Thus, the difference in expression between radial positions where the gene is most and least expressed cannot exceed 10^{-3}. We find that this prior helps stabilize numerical parameter optimization, in particular for genes with too little UMIs to identify spatial regulation with high confidence levels.

Numerically, we optimize $L := \log P(n_{gc}, r_c | \alpha, \beta, \gamma, \sigma) + \log P(\alpha, \beta, \gamma)$ for $\alpha, \beta, \gamma, \sigma$ one gene at a time using the Nedler-Mead algorithm implemented in the `optim()` function of the R software. To facilitate stable convergence to optimal likelihood, we first maximize L under the constraints $\gamma = 0$ (no quadratic trend, only linear trend) and $\sigma = 1$ (Poisson distribution). From the resulting optimal (α, β) as a starting point, we then optimize the full parameter set

$(\alpha, \beta, \gamma, \sigma)$ using the same approach. Simulating gene expression using the best-fitted negative binomial distribution produces spatial UMI counts that faitfully mimick UMI counts observed in the data (Fig. 2).

Only genes with an average of at least 1 UMI per cell are used for inference: genes with an average of less than 1 UMI per cell provide too little data to estimate spatial regulation.

To obtain confidence intervals on the parameters α, β, γ, we compute the Hessian of the log-likelihood for the best fitting parameters. The inverse square root of the diagonal terms of the Hessian estimates the standard deviation of the parameters — the uncertainty on these parameters — following Wilks' theorem [4].

The standard deviation of the parameters estimated in this fashion is used to compute a p-value to test that the quadratic and linear trends of each gene is non-zero. For the quadratic trend γ, for example,

$$p = 2\left(1 - \Phi(|\gamma|/\sigma_\gamma)\right)$$

with $\Phi(x)$ the cumulative standard Gaussian distribution function. The factor 2 accounts for bi-lateral testing of upward or downward trends.

To control for the false discovery rate (fdr) in multiple hypothesis testing, we apply the Benjamini-Hochberg correction to the concatenation of all p-values from all genes. Spatial genes are defined as genes with a log likelihood $L > -7500$ (following visual examination of the goodness of fit of genes with different log likelidhoo, Fig. S2a), a fdr < 10% for either linear or quadratic trends, and at least a 30% difference in gene expression across radial positions.

1.2 Classifying genes' spatial expression pattern

We classify genes into core, peripheral, intermediate and extrema spatial expression patterns based on the spatial regulation function $f_g(r) = a + br + cr^2$ inferred for each gene g. For a *core*-expressed gene g, $f_g(r)$ is maximal in the core $r = 0$ and decreases with increasing r. Therefore,

$$\frac{df}{dr} < 0, r \in [0,1] \Leftrightarrow b < 0 \wedge b + 2c < 0.$$

Conversely, for a *peripheral* gene,

$$\frac{df}{dr} > 0, r \in [0,1] \Leftrightarrow b > 0 \wedge b + 2c > 0.$$

We define a gene expressed in *intermediate* layers as a gene whose expression peaks at a radial position $1/3 < r^* < 2/3$

$$\left.\frac{df}{dr}\right|_{r=r^*} = 0, \left.\frac{df^2}{dr^2}\right|_{r=r^*} < 0, 1/3 < r^* < 2/3$$

which implies

$$c < 0 \wedge 1/3 < -b/2c < 2/3.$$

Conversely, we define an *extrema*-expressed gene as a gene with minimal expression at a radial position $1/3 < r^* < 2/3$

$$\left.\frac{df}{dr}\right|_{r=r^*} = 0, \left.\frac{df^2}{dr^2}\right|_{r=r^*} > 0, 1/3 < r^* < 2/3$$

which implies

$$c > 0 \wedge 1/3 < -b/2c < 2/3.$$

References

1. Raj, A., Peskin, C. S., Tranchina, D., Vargas, D. Y. & Tyagi, S. Stochastic mRNA synthesis in mammalian cells. *PLoS biology* **4**, e309, DOI: 10.1371/journal.pbio.0040309 (2006). Publisher: Public Library of Science.

2. Shahrezaei, V. & Swain, P. S. Analytical distributions for stochastic gene expression. *Proc. Natl. Acad. Sci. United States Am.* **105**, 17256–61, DOI: 10.1073/pnas.0803850105 (2008). ArXiv: 0812.3344 ISBN: 1091-6490 (Electronic)\n0027-8424 (Linking).

3. Breda, J., Zavolan, M. & van Nimwegen, E. Bayesian inference of gene expression states from single-cell RNA-seq data. *Nat. Biotechnol.* **39**, 1008–1016, DOI: 10.1038/s41587-021-00875-x (2021). Publisher: Nature Publishing Group.

4. Sivia, D. & Skilling, J. Data Analysis: A Bayesian Tutorial. *Technometrics* **40**, 155, DOI: 10.2307/1270652 (1998). ISBN: 0198568320.
