# Supplementary material for: Diffusion Smart-seq3 of breast cancer spheroids to explore spatial tumor biology and test evolutionary principles of tumor heterogeneity
Source: Sci Rep. 2025 Jan 30;15:3811. doi: 10.1038/s41598-024-83989-x (PMC11782488; doi:10.1038/s41598-024-83989-x)
Supplement: Supplementary file 2 — Supplementary Material 2 [file 41598_2024_83989_MOESM2_ESM.docx]

# Supplementary figures


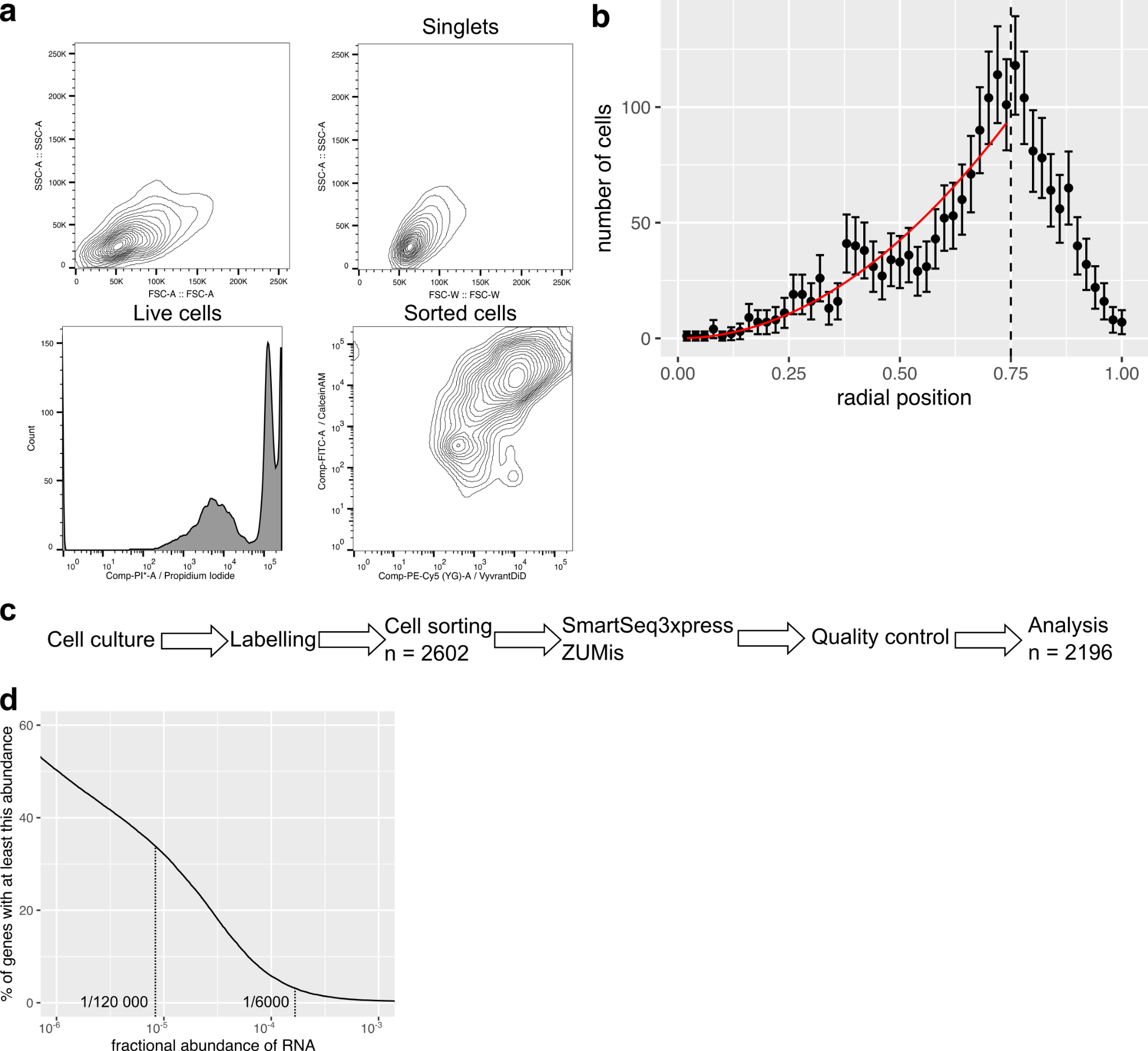


**Figure S1:** **a.** Gating strategy to isolate single, live cells and joint distribution of Vybrant-DiL and Calcein-AM intensities. See Methods - Flow Cytometry for details. **b.** Number of sequenced cells as a function of radial position inferred from Calcein-AM intensity. The number of cells increases quadratically with the radial position, as expected given the spherical geometry of spheroids, since the surface area and thus the number of cells grows quadratically with *r*. There are less cells expected at radial positions > 0.75. Possible reasons for this include: a lower cellular density and higher extracellular matrix content at the spheroid periphery, peeling of the outermost layers of spheroids during washing steps prior to dissociation, and a higher proportion of dead cells among the spheroid periphery. **c.** Workflow steps including the number of cells isolated and passing the quality controls. **d.** Reverse cumulative distribution of fractional mRNA abundance. RNAs from 3% of genes have a fractional abundance of at least 1/6000th, and RNAs from 34% of genes have a fractional abundance of at least 1/120,000th. RNA abundance was estimated by pulling Smart-seq3xpress UMIs from 1000+ tumor spheroid cells (Methods).


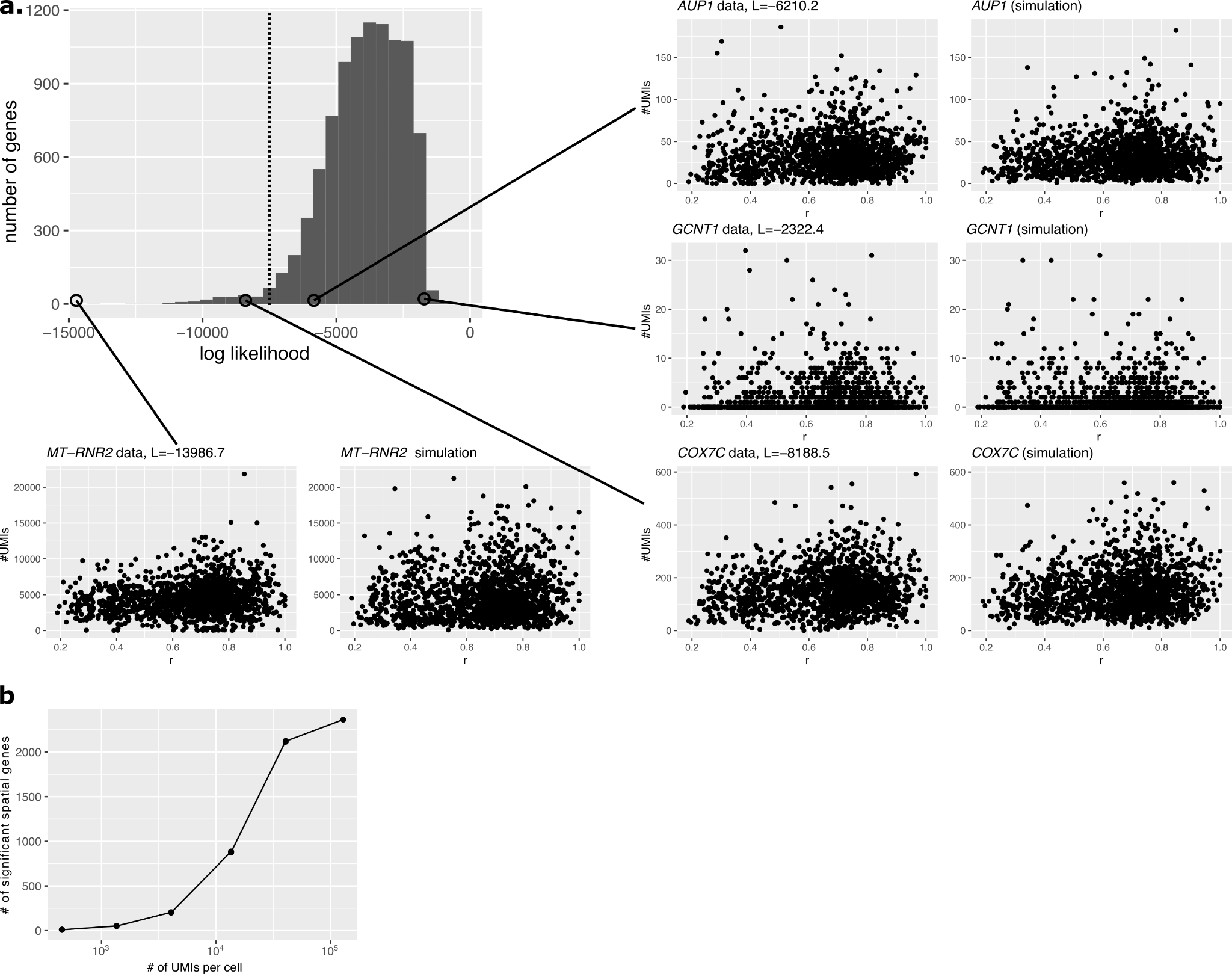


**Figure S2: a.** Most genes fit the single-cell spatial stochastic model of gene expression well. Histogram: distribution of log likelihood for all fitted genes. Scatter plot: data vs simulated UMI counts (y-axis, *ngc:* #UMIs for gene *g* in cell *c*) vs radial position (x-axis, *r*) for four genes with different likelihoods. Dots represent single cells. **b.** Sequencing more RNAs per cell is unlikely to identify many more spatial genes efficiently: down-sampling UMI counts per cell reveals that the number of identified spatial genes follows a saturating sigmoid curve.


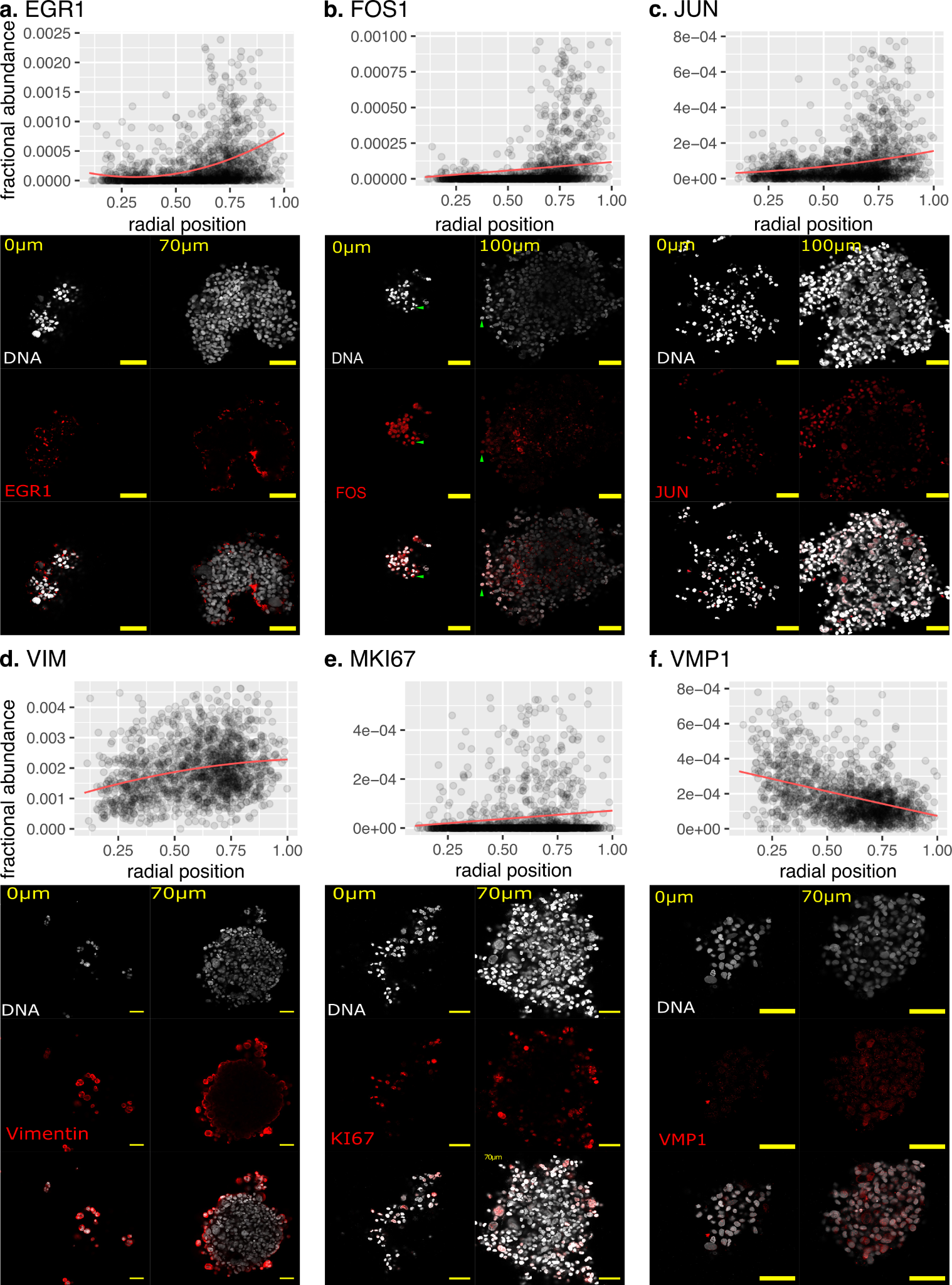


**Figure S3:** Spatial gene regulation inferred by Smart-Seq3D is validated by immunofluorescence stainings. Radial distribution of selected transcripts abundance (upper panel) and immunostaining of the corresponding protein (red) in spheroids counterstained for DNA with Hoechst 33342 (white, lower panel). **a.** EGR1. **b.** FOS. **c.** JUN. **d.** VIM. **e.** MKI67. **f.** VMP1. Scale bar: 100 um. The depth of the confocal imaging plane appears on the top left of each microscopy panel: 0um = spheroid surface, 70-100um = inside the spheroid.


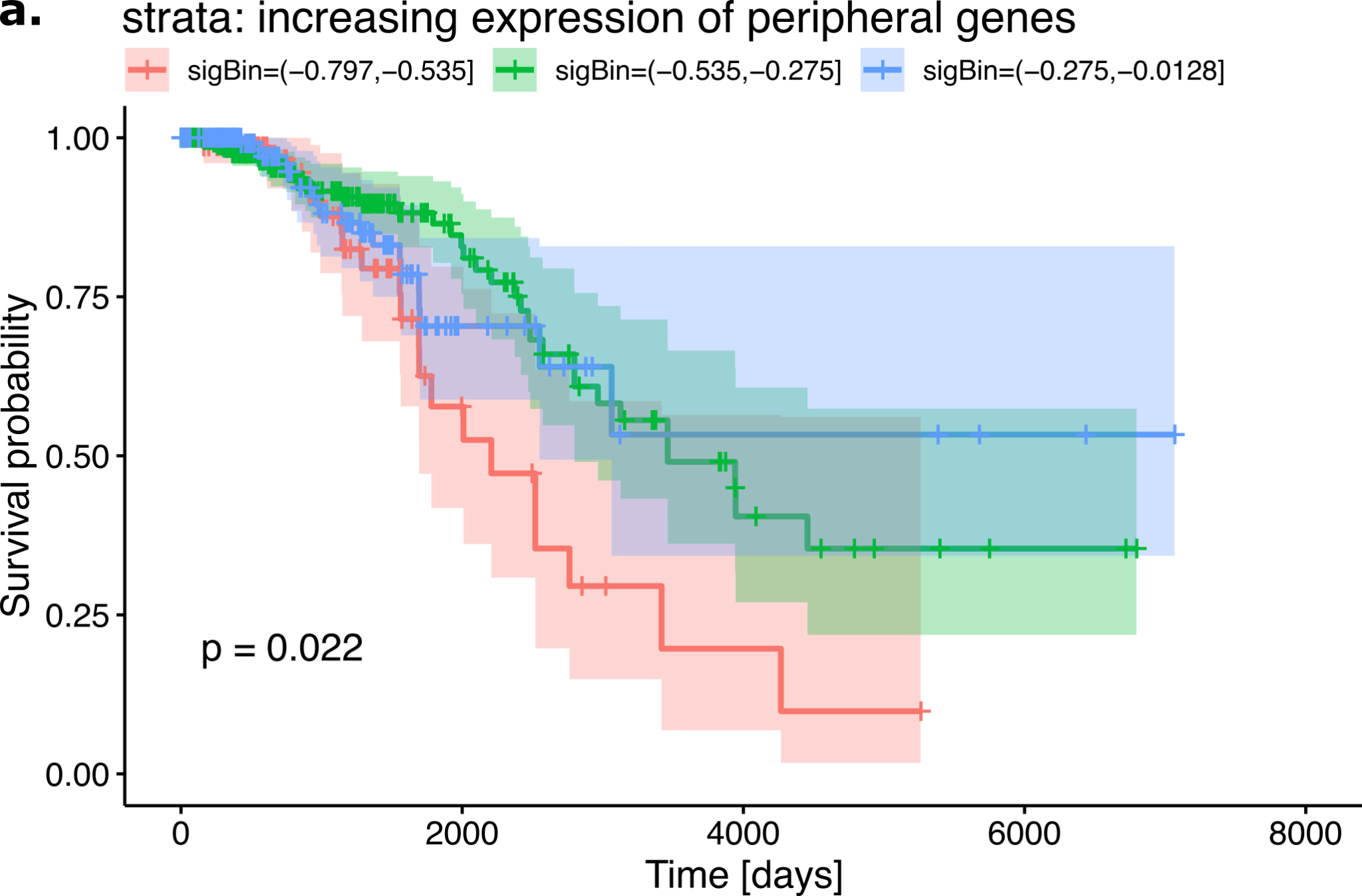


**Figure S4: a.** Higher expression of peripheral genes associates with longer survival in patients of the TCGA breast cohort. Kaplan-meier estimate of overall survival probability was computed for patients with low, average and high expression of peripheral genes. P-value: log-rank test.


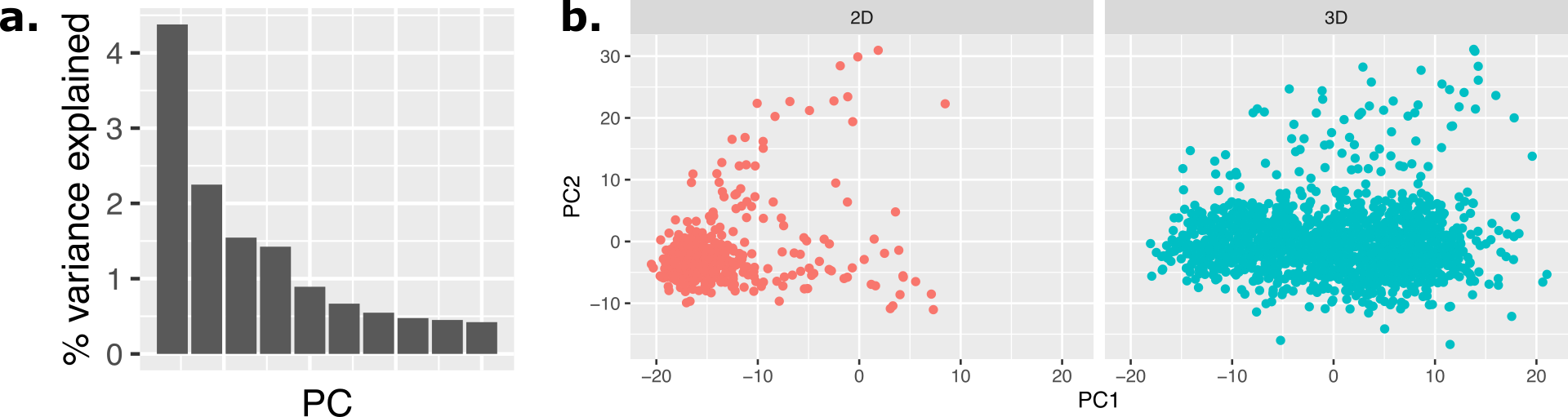


**Figure S5: a.** The first principal component of tumor spheroid single-cell gene expression captures 1.9x more variance than the second principal component, suggesting a 1-dimensional transcriptional heterogeneity. **b.** Cells grown in 2D fall on one end of the 1D continuum of transcriptional heterogeneity of cells grown in 3D. The transcriptomes of single MDA-MB-231 cells grown in 2D were projected on the first two principal components of the transcriptomes of MDA-MB-231 cells grown in 3D spheroids.


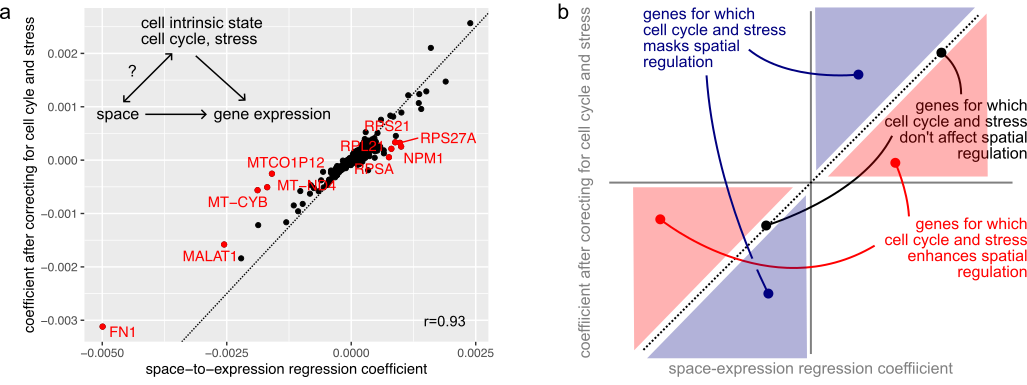


**Figure S6:** Controlling for cell cycle and stress has little impact on spatial - gene expression associations **a.** The slopes of gene-wise space-to-expression functions are similar when correlating space and expression directly and when regressing out cell cycle and stress genes. **b**. Guide to interpret panel a. Genes for which cell cycle and stress don’t affect spatial regulation have equal slope in the slope estimated by univariate regression of expression on space, and in the multi-variate regression of expression on space, cell cycle and stress. Genes for which cell cycle and stress enhance or mask spatial regulation lie closer to the x- and y-axes, respectively.
